# Secondary Cases of Invasive Disease Caused by Encapsulated and Nontypeable *Haemophilus influenzae* — 10 U.S. Jurisdictions, 2011–2018

**DOI:** 10.15585/mmwr.mm7215a2

**Published:** 2023-04-14

**Authors:** Sara E. Oliver, Amy B. Rubis, Heidi M. Soeters, Arthur Reingold, Meghan Barnes, Susan Petit, Ashley E. Moore, Lee H. Harrison, Ruth Lynfield, Kathy M. Angeles, Kari E. Burzlaff, Ann Thomas, William Schaffner, Henju Marjuki, Xin Wang, Susan Hariri

**Affiliations:** ^1^National Center for Immunization and Respiratory Diseases, CDC; ^2^School of Public Health, University of California, Berkeley, Berkeley, California; ^3^Colorado Department of Public Health & Environment; ^4^Connecticut Department of Public Health; ^5^Georgia Department of Public Health; ^6^Johns Hopkins Bloomberg School of Public Health, Baltimore, Maryland; ^7^Minnesota Department of Health; ^8^New Mexico Department of Health; ^9^New York State Department of Health; ^10^Oregon Health Authority; ^11^Vanderbilt University School of Medicine, Nashville, Tennessee.

*Haemophilus influenzae* (Hi) can cause meningitis and other serious invasive disease. Encapsulated Hi is classified into six serotypes (a–f) based on chemical composition of the polysaccharide capsule; unencapsulated strains are termed nontypeable Hi (NTHi). Hi serotype b (Hib) was the most common cause of bacterial meningitis in children in the pre-Hib vaccine era, and secondary transmission of Hi among children (e.g., to household contacts and in child care facilities) (*1*,*2*) led to the Advisory Committee on Immunization Practices (ACIP) recommendation for antibiotic chemoprophylaxis to prevent Hib disease in certain circumstances.* High Hib vaccination coverage since the 1990s has substantially reduced Hib disease, and other serotypes now account for most Hi-associated invasive disease in the United States (*3*). Nevertheless, CDC does not currently recommend chemoprophylaxis for contacts of persons with invasive disease caused by serotypes other than Hib and by NTHi (non-b Hi). Given this changing epidemiology, U.S. surveillance data were reviewed to investigate secondary cases of invasive disease caused by Hi. The estimated prevalence of secondary transmission was 0.32% among persons with encapsulated Hi disease (≤60 days of one another) and 0.12% among persons with NTHi disease (≤14 days of one another). Isolates from all Hi case pairs were genetically closely related, and all patients with potential secondary infection had underlying medical conditions. These results strongly suggest that secondary transmission of non-b Hi occurs. Expansion of Hi chemoprophylaxis recommendations might be warranted to control invasive Hi disease in certain populations in the United States, but further analysis is needed to evaluate the potential benefits against the risks, such as increased antibiotic use.

Before the introduction of Hib vaccines in the 1980s, Hib was the most common cause of bacterial meningitis in the United States, accounting for 95%–98% of all cases of invasive Hi disease (*4*). Studies during the pre-Hib vaccine era documented high prevalence of Hib colonization as well as secondary transmission among children exposed to Hib in a household or child care facility setting (*1*,*2*). Reported risk for secondary disease ranged from 1.2% in children aged 12–23 months (*2*) to 6% in infants aged <12 months (*1*). ACIP recommended antibiotic chemoprophylaxis in selected circumstances to prevent secondary Hib transmission. Since licensure and recommendation for Hib vaccines were implemented, the incidence of invasive Hib disease in the United States has declined by approximately 99%, accounting for only 1.3% of invasive Hi disease in 2018. However, invasive disease caused by non-b Hi, particularly serotype a (Hia) and NTHi, has been increasing. During 2008–2017, the overall incidence of Hia increased by 11.1% annually in the United States (*5*). Despite the changing epidemiology of Hi disease, ACIP recommendations for prevention and control of Hib disease in the United States published in 2014 stated that chemoprophylaxis is not recommended for prophylaxis against cases of invasive disease caused by non-b Hi, because secondary transmission has not been documented. Data collected as part of an active, population-based surveillance network were analyzed to investigate possible instances of secondary transmission of Hi.

Cases of invasive Hi disease were identified through Active Bacterial Core surveillance in 10 U.S. jurisdictions.^†^ Clusters of encapsulated Hi disease were defined as cases of invasive disease caused by the same serotype diagnosed in the same county that occurred ≤60 days of one another during 2011–2018. Clusters of unencapsulated Hi disease were defined as cases of invasive NTHi disease in the same county that occurred ≤14 days of one another during 2015–2018; the restricted periods were selected because of the high incidence of NTHi and limited resources. To identify potential secondary transmission among clusters, site personnel reviewed information collected as part of the public health case investigations; patients were not recontacted for this study. Cases were only reviewed through 2018, and the data presented in this analysis is the only available data. Within each cluster, potential secondary transmission was defined as the occurrence of two or more confirmed or suspected epidemiologically linked cases. Pairs of NTHi cases occurring in a mother and infant aged <30 days were excluded from this analysis; the infant cases in these pairs occurred in the first day of life and might have resulted from intrauterine perinatal transmission. If secondary transmission was suspected, whole genome sequencing (WGS) of patient isolates was conducted to evaluate sequence relatedness through single nucleotide polymorphism (SNP) differences. Although no formal threshold for identification of related isolates exists, for this analysis, isolate pairs with fewer than 10 SNP differences were considered closely related, based on a previous analysis of Hi genetic diversity (*6*). Secondary transmission prevalence was calculated as the number of likely secondary cases divided by the total number of reported cases, expressed as a percentage. This activity was reviewed by CDC and was conducted consistent with applicable federal law and CDC policy.^§^

Among 1,584 cases of encapsulated invasive Hi disease reported during 2011–2018, a total of 157 clusters with five instances of likely secondary transmission were identified, for an estimated secondary transmission prevalence among encapsulated Hi cases of 0.32% within 60 days of one another (Table 1). Three case pairs were Hi serotype f, one pair was Hia, and one pair was Hi serotype e; no secondary Hib case pairs occurred. Among 2,426 cases of NTHi disease reported during 2015–2018, a total of 373 clusters with three instances of likely secondary transmission were identified, for an estimated prevalence of secondary transmission among NTHi cases of 0.12% within 14 days of one another. All isolates from possible secondary cases had 0–1 SNP differences from the primary case isolates, indicating the isolates were genetically highly related.

**TABLE 1:** *Haemophilus influenzae* cases, clusters, and pairs of secondary transmission, by serotype — Active Bacterial Core surveillance, 10 U.S. jurisdictions,* 2011–2018

Hi type	Reported cases, no.	Possible clusters,^†^ no.	Pairs of secondary transmission,^§^ no.	Prevalence of secondary transmission, %
**Serotype (encapsulated Hi)**				
a	366	20	1	0.27
b	87	6	0	—
c	0	—	—	—
d	5	0	0	—
e	261	19	1	0.38
f	865	112	3	0.35
All encapsulated Hi^¶^	1,584	157	5	0.32
NTHi (unencapsulated)**	**2,426**	**373**	**3**	**0.12**

**Abbreviations**: Hi = *Haemophilus influenzae*; NTHi = nontypeable Hi.

* California (three San Francisco Bay Area counties), Colorado (five Denver-area counties), Connecticut (statewide), Georgia (statewide), Maryland (statewide), Minnesota (statewide), New Mexico (statewide), New York (15 Rochester- and Albany-area counties), Oregon (statewide), and Tennessee (20 counties).

^†^ Cases of the same serotype occurring in the same county ≤60 days of one another for encapsulated Hi cases and ≤14 days of one another for cases of NTHi.

**^§^** Confirmed or suspected epidemiologic link between cases and less than 10 single nucleotide polymorphism differences.

^¶^ Cases occurring during 2011–2018 were reviewed.

** Cases occurring during 2015–2018 were reviewed.

Among five instances of secondary transmission of encapsulated Hi, epidemiologic links identified were in 1) household family contacts (three pairs), 2) residents in the same long-term care facility (one pair), and 3) persons experiencing homelessness (one pair, admitted to the same hospital 11 days apart) (Table 2). Among three instances of likely secondary transmission of NTHi, two pairs occurred among residents of the same long-term care facility and one occurred in residents of the same household (Table 3). All eight likely secondary cases (encapsulated and nontypeable) were diagnosed ≤2 weeks after the primary case, with six occurring ≤7 days after the primary case. All likely secondary cases occurred in patients reported to have an underlying medical condition, and all but one occurred in adults.

**TABLE 2:** Epidemiologic and clinical characteristics of five pairs of secondary transmission of encapsulated *Haemophilus influenzae* within 60 days of one another — Active Bacterial Core surveillance, 10 U.S. jurisdictions,* 2011–2018

Characteristic	Pair 1	Pair 2	Pair 3	Pair 4	Pair 5
Serotype	Hia	Hie	Hif	Hif	Hif
Year of diagnosis	2014	2017	2011	2012	2017
Epidemiologic link^†^	Son/Mother	Mother/Son	Twins	Reside at same LTCF	Both experiencing homelessness
Sex and age	Male, 15 yrs; female, 56 yrs	Female, 59 yrs; male, 32 yrs	Male, 8 mos; male, 8 mos	Female, 79 yrs; male, 88 yrs	Male, 50 yrs; male, 48 yrs
Days between positive cultures	6	4	1	7	11
Clinical syndrome, primary patient	Bacteremic pneumonia	Bacteremic pneumonia	Meningitis, empyema, and septic arthritis	Bacteremic pneumonia	Meningitis
Clinical syndrome, secondary patient	Bacteremic pneumonia	Bacteremic pneumonia	Bacteremia and septic arthritis	Bacteremic pneumonia	Bacteremic pneumonia
Underlying medical conditions, primary patient	None	Peripheral vascular disease and substance abuse	None	COPD and dementia	Asplenia
Underlying medical conditions, secondary patient	Diabetes and obesity	Neuromuscular disorder and seizure disorder	Chronic skin breakdown	Renal insufficiency and dementia	Stroke, cirrhosis, COPD, seizure disorder, and substance abuse
Outcomes	Both survived	Both survived	Both survived	Both survived	Both survived
Sequence type	ST-56	ST-18	ST-124	ST-124	ST-124
SNP difference	1	1	0	1	0

**Abbreviations:** COPD = chronic obstructive pulmonary disease; Hia = Hi serotype a; Hie = Hi serotype e; Hif = Hi serotype f; LTCF = long-term care facility; SNP = single nucleotide polymorphism; ST = sequence type.

* California (three San Francisco Bay Area counties), Colorado (five Denver-area counties), Connecticut (statewide), Georgia (statewide), Maryland (statewide), Minnesota (statewide), New Mexico (statewide), New York (15 Rochester- and Albany-area counties), Oregon (statewide), and Tennessee (20 counties).

^†^ The primary patient is listed first, and the secondary patient is listed second.

**TABLE 3:** Epidemiologic and clinical characteristics of three pairs of secondary transmission of nontypeable *Haemophilus influenzae* within 14 days of one another — Active Bacterial Core surveillance, 10 U.S. jurisdictions,* 2015–2018

Characteristic	Pair 1	Pair 2	Pair 3
Year of diagnosis	2015	2017	2017
Epidemiologic link	Reside at same LTCF	Reside at same LTCF	Reside in same household
Sex and age^†^	Male, 68 yrs; male, 85 yrs	Female, 81 yrs; female, 94 yrs	Female, 88 yrs; female, 81 yrs
Days between positive culture results	2	9	2
Clinical syndrome, primary patient	Bacteremic pneumonia	Bacteremic pneumonia	Bacteremic pneumonia
Clinical syndrome, secondary patient	Bacteremic pneumonia	Bacteremic pneumonia	Bacteremic pneumonia
Underlying medical conditions, primary patient	COPD, congestive heart failure, diabetes, and obesity	Chronic kidney disease, congestive heart failure, dementia, and obesity	ACVD, dementia, and stroke
Underlying medical conditions, secondary patient	ACVD, chronic kidney disease, peripheral neuropathy, solid organ malignancy, and stroke	ACVD and obesity	ACVD, diabetes, and stroke
Outcomes	Primary patient survived; secondary patient died	Primary patient survived; secondary patient died	Primary patient died; secondary patient survived
Sequence type	ST-165	ST-142	ST-142
SNP difference	0	1	0

**Abbreviations**: ACVD = atherosclerotic cardiovascular disease; COPD = chronic obstructive pulmonary disease; LTCF = long-term care facility; NTHi = nontypeable *Haemophilus influenzae*; SNP = single nucleotide polymorphism; ST = sequence type.

* California (three San Francisco Bay Area counties), Colorado (five Denver-area counties), Connecticut (statewide), Georgia (statewide), Maryland (statewide), Minnesota (statewide), New Mexico (statewide), New York (15 Rochester- and Albany-area counties), Oregon (statewide), and Tennessee (20 counties).

^†^ The primary patient is listed first, and the secondary patient is listed second.

## Discussion

Since Hib vaccine became available in the 1980s, most invasive Hi disease in the United States has been caused by non-b serotypes or nontypeable strains. Although this study found no evidence of secondary transmission among Hib clusters, possibly reflecting the effectiveness of vaccination and existing chemoprophylaxis recommendations to prevent secondary Hib infection, the findings do suggest that secondary transmission of non-b Hi likely occurs in the United States in a small percentage of cases. In all instances of likely secondary transmission of non-b Hi, the second patient had one or more underlying medical conditions that might have predisposed them to invasive infections.

Secondary transmission is not routinely assessed as part of national Hi surveillance. Other than the present analysis, the only data on possible secondary transmission of non-b Hi in the United States are from a 2019 report of two infants with Hia meningitis who attended the same child care facility in Texas and were admitted to the hospital ≤17 days of one another (*7*). WGS conducted at CDC revealed that both isolates were sequence type 576 with no SNP differences. Although no secondary cases were identified during a 2018 Hia outbreak in a small rural Alaskan community, an evaluation found that nasopharyngeal carriage of Hia was highest among close contacts, and no further cases occurred after administration of chemoprophylaxis (*8*).

Given the increasing incidence of non-b Hi disease and the occurrence of Hia outbreaks in some communities in the United States, chemoprophylaxis has been recommended for close contacts of Hia cases by jurisdictions with high Hia disease incidence. In 2018, the Alaska Department of Health and Social Services recommended that clinicians strongly consider offering chemoprophylaxis to close contacts of patients with invasive Hia, particularly when there are household contacts who are aged <4 years or who are immunocompromised.^¶^ This recommendation is similar to those adopted in tribal communities in the southwest United States that experience an elevated incidence of invasive disease caused by Hia and Hib (*9*). In addition, since 2018, the Committee on Infectious Diseases of the American Academy of Pediatrics recommends that clinicians consider prophylaxis for cases of invasive Hia disease in households with children aged <4 years or children who are immunocompromised and recommends a similar approach to child care facility contacts in consultation with public health officials (*10*).

The findings in this study are subject to at least three limitations. First, prevalence of secondary transmission was likely underestimated because epidemiologic connections were established using only routinely collected data, and WGS was not performed on all isolates. Second, prevalence of secondary NTHi is likely further underestimated because the cluster definition was limited to cases that occurred within 14 days of one another, whereas 60 days is usually used for defining secondary Hi. Additional studies are needed to evaluate secondary cases of NTHi that occurred within 60 days of one another. Finally, data were only available for this analysis through 2018. Although these data might not fully reflect current Hi epidemiology, these findings strongly suggest secondary transmission of non-b Hi occurs in the United States and are relevant to guide updates to current chemoprophylaxis recommendations.

Invasive Hi disease is serious and can be life-threatening. Chemoprophylaxis taken by close contacts is effective in preventing secondary transmission of Hib and might be an important tool for preventing secondary cases of non-b Hi disease. Given the changing epidemiology of Hi in the United States and likely secondary transmission of non-b Hi documented in this report, expanding the current chemoprophylaxis recommendations might be warranted to prevent disease in certain populations and might facilitate clinical and public health decision-making, especially because chemoprophylaxis must often be offered before serotyping results are available. Further analysis is needed to evaluate the potential benefits of changing chemoprophylaxis recommendations for Hi against the risks, such as increased antibiotic use.

**Summary**

**What is already known about this topic?**

Widespread vaccination has reduced invasive disease caused by *Haemophilus influenzae* (Hi) type b in the United States by approximately 99%, but incidence of disease caused by non-type b Hi has been increasing. CDC does not currently recommend chemoprophylaxis for contacts of persons with invasive disease caused by non-type b Hi.

**What is added by this report?**

Analysis of Active Bacterial Core surveillance from 10 U.S. jurisdictions identified eight instances of likely secondary transmission of non-type b Hi, all among patients with underlying medical conditions.

**What are the implications for public health practice?**

These results strongly suggest that secondary transmission of non-type b Hi occurs. Expansion of Hi chemoprophylaxis recommendations might be warranted to control invasive Hi disease in certain populations in the United States, but further analysis is needed to evaluate the potential benefits against the risks, such as increased antibiotic use.

## Footnotes

* Rifampin chemoprophylaxis is recommended for index patients (unless index patients are treated with cefotaxime or ceftriaxone, both of which eradicate Hib colonization) and all household contacts in households with members aged <4 years who are not fully vaccinated or members aged <18 years who are immunocompromised, regardless of their vaccination status. Chemoprophylaxis is recommended in child care facility settings when two or more cases of invasive Hib disease have occurred ≤60 days of one another and unimmunized or underimmunized children attend the facility. https://www.cdc.gov/mmwr/preview/mmwrhtml/rr6301a1.htm

† California (three San Francisco Bay Area counties), Colorado (five Denver-area counties), Connecticut (statewide), Georgia (statewide), Maryland (statewide), Minnesota (statewide), New Mexico (statewide), New York (15 Rochester- and Albany-area counties), Oregon (statewide), and Tennessee (20 counties).

§ 45 C.F.R. part 46.102(l)(2), 21 C.F.R. part 56; 42 U.S.C. Sect. 241(d); 5 U.S.C. Sect. 552a; 44 U.S.C. Sect. 3501 et seq.

¶
http://www.epi.alaska.gov/bulletins/docs/b2018_09.pdf
